# Understanding Glioblastoma Biomarkers: Knocking a Mountain with a Hammer

**DOI:** 10.3390/cells9051236

**Published:** 2020-05-16

**Authors:** Malak Hassn Mesrati, Amir Barzegar Behrooz, Asmaa Y. Abuhamad, Amir Syahir

**Affiliations:** Department of Biochemistry, Faculty of Biotechnology and Biomolecular Sciences, Universiti Putra Malaysia, UPM Serdang 43400, Selangor, Malaysia; malak.hassan.rh@gmail.com (M.H.M.); am.barzegar.behrooz@gmail.com (A.B.B.); abuhamadasmaa@gmail.com (A.Y.A.)

**Keywords:** gliomas, glioblastoma, cancer stem cells, CSCs markers, miRNAs

## Abstract

Gliomas are the most frequent and deadly form of human primary brain tumors. Among them, the most common and aggressive type is the high-grade glioblastoma multiforme (GBM), which rapidly grows and renders patients a very poor prognosis. Meanwhile, cancer stem cells (CSCs) have been determined in gliomas and play vital roles in driving tumor growth due to their competency in self-renewal and proliferation. Studies of gliomas have recognized CSCs via specific markers. This review comprehensively examines the current knowledge of the most significant CSCs markers in gliomas in general and in glioblastoma in particular and specifically focuses on their outlook and importance in gliomas CSCs research. We suggest that CSCs should be the superior therapeutic approach by directly targeting the markers. In addition, we highlight the association of these markers with each other in relation to their cascading pathways, and interactions with functional miRNAs, providing the role of the networks axes in glioblastoma signaling pathways.

## 1. Introduction

**Figure 1:**
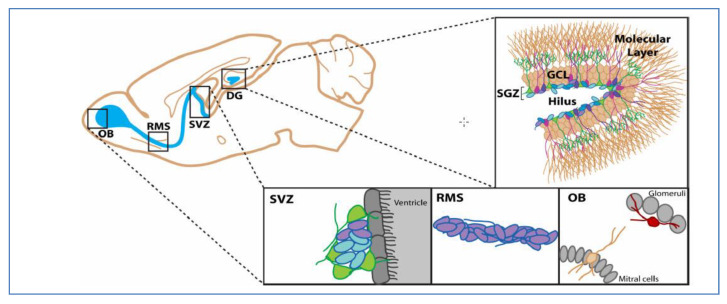
Candidate regions of cells of origin of gliomas in adult mouse brain (a sagittal view). Blue: neurogenic area, Green: stem cells, Purple: neuroblasts, RMS: Rostral Migratory Stream, OB: Olfocatory Bulb, DG: Dentate Gyrus, GCL: Granule Cell Layer, SVZ: Subventricular Zone and SGZ: Subgranular Zone. (Reproduced from Johnson et al. [7] with permission from the corresponding author).

In the early 19th century, Durante and Conheim proposed the idea of cancer cells’ formation from stem cells. According to the embryonal rest theory of cancer that was established by these two scientists, the remnants of embryonic components remain in the tissues of adult organs [1]. After the introduction of this theory, various experiments were carried out and in the late 1990s for the first time; Bonnet and Dick identified cancer stem cells in acute myeloid leukemia [2]. CSCs have been recognized in diverse types of tumors, including the lung, colon, breast, prostate, and brain [3]. The presence of stem-like cells in brain tumors was initially identified in 2002 by Ignatova and his colleagues [4]. Although the existence of stem cells in brain tumors has been identified, the cell of origin of glioma is still a debatable question. Several reports have shown that brain stem cells are caused by dedifferentiation of brain cells or transformation of progenitor cells [3]. In the adult brain of mammalians, there are two specific neurogenic regions: 1) Subventricular zone (SVZ) in the forebrain lateral ventricles, and 2) subgranular zone (SGZ) in the dentate gyrus of the hippocampus, in which quiescent stem cells and activated progenitor cells are located [3,5]. According to previous findings, SVZ is most likely to be the region of the cell of origin of gliomas [6,7] and perhaps the region where gliomas stem cells markers exist (Figure 1).

The World Health Organization (WHO) has proposed two types of glioma classifications, which were in the year 2007 and 2016. The former was based on histological criteria, and glioma tumors were divided into three categories: Astrocytoma, oligodendroglioma, and mixed oligoastrocytoma. Tumor malignancy was graded from II–IV in accordance with morphological criteria. However, due to obstacles, such as high interobserver variability and survival variation within the grades, neuropathologist and glioma biologists sought other markers to enhance the characterization of clinically relevant subgroups [8,9,10]. In 2016, WHO consolidated tumor morphology, *IDH* mutation, and 1p19q co-deletion status into a new classification system for adult diffuse glioma. Accordingly, adult diffuse glioma was divided into five categories: Glioblastoma *IDH*-wild type, glioblastoma *IDH*-mutant, diffuse or anaplastic astrocytomas with *IDH*-wild type and its *IDH*-mutant type, and oligodendroglioma or anaplastic oligodendroglioma with *IDH*-mutant along with 1p19q co-deletion [11,12]. Glioblastoma is by far the commonest and the most malignant type of diffuse gliomas and is associated with poor prognosis, with a medium patient survival of 12–15 months [13]. *IDH*-wildtype glioblastoma, which corresponds closely to primary glioblastoma, mainly arises in patients over the age of 55 years whereas *IDH*-mutant glioblastoma, which corresponds to secondary glioblastoma, predominates in younger patients [14]. 

Recently, the putative markers frequently being used to identify and/or isolate glioblastoma stem cells (GSCs) include: CD133, CD44, CD15, CD70 (CD27 L), S100A4, ALDH1A3, Nanog, OCT-4, SOX-2, and Nestin. A deeper understanding of the roles of these markers as well as their engagements in many crucial cellular signaling pathways can guide promising research towards novel GBM treatments. Here, we summarize the studies on the role of the most common GSCs biomarkers in mediating tumorigenesis and tumor resistance along with recent advances in targeting GSCs markers as a putative therapeutic strategy. Moreover, we review recent advances in the development of specific and sensitive biomarkers, such as microRNAs (miRNAs), for cancer diagnosis and prognosis. We attempt to provide a comprehensive profile of key biomarker-biomarker and biomarkers-miRNAs involved in stemness, proliferation, migration, invasion, therapeutic resistance, and survival in GBM.

## 2. Putative Biomarkers of Glioblastoma Stem Cells 

### 2.1. CD133 

CD133 (also known as Prominin-1) is encoded by the *PROM1* gene [15] and localizes to membrane protrusions of normal and cancer cells [16]. *PROM1*, located on chromosome 4p15, includes 37 exons, and is translated into a 120 kDa glycoprotein with five transmembrane domains [17,18]. Several studies have identified CD133 as a prominent CSCs marker associated with cancer progression and tumorigenesis in various tumors, including gliomas, colorectal cancer, pancreatic cancer, ovarian cancer, prostate carcinoma, and hepatocellular carcinoma [18,19]. 

In glioblastoma, overexpression of CD133 has been excessively linked to CSCs’ self-renewal and resistance towards temozolomide (TMZ) by the activation of c-Jun N-terminal kinase (JNK) signaling [20] and Notch/sonic hedgehog (SHH) pathways, respectively [21]. In addition, CD133^Pos^ cells populations were found to be highly associated with aldehyde dehydrogenase 3A1 (ALDH3A1), another putative marker that promotes poor prognosis and chemoresistance [22,23]. ALDH3A1 expression in gliomas was shown to be modulated by Wingless (Wnt) or the Wnt/β-catenin signaling pathway [22], one of the deep-rooted signaling pathways during tumorigenesis that can be blocked by pharmacological meddling of LGK974 in glioblastoma cells [24,25]. Suwala et al. [22] demonstrated that LGK974-mediated Wnt pathway suppression reduced the expression of CD133 and enhanced the efficacy of TMZ via the inhibition of ALDH3A1. Moreover, Li et al. showed that glioblastomas expressing high levels of CD133 are extremely correlated with the expression of HOX gene stem cell factors, a prognostic marker found to be associated with survival and chemotherapeutic resistance. Neither the overexpression of HOX genes nor CD133 alone was adequate to drive glioblastoma progression, whereas overexpression of both worsened its prognosis [15]. 

**Figure 2:**
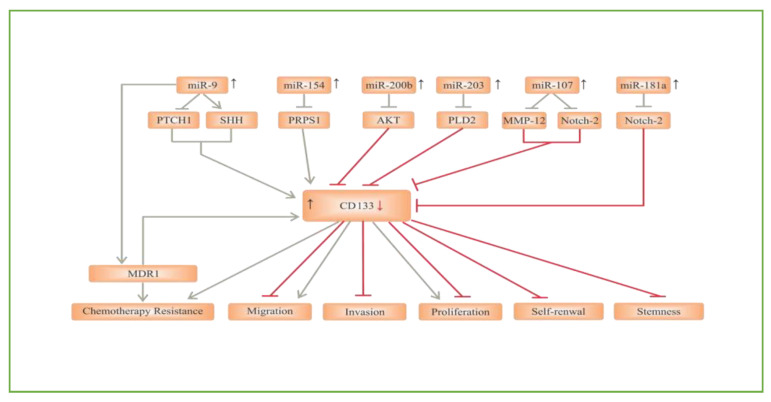
The role of miRNAs/CD133 axis in glioblastoma signaling pathways. Phospholipase D2 (PLD2), Phosphoribosyl pyrophosphate synthetase 1 (PRPS1), Protein patched homolog 1 (PTCH1), Sonic Hedgehog signaling pathway (SHH), Multidrug resistance protein 1 (MDR1), Matrix metalloproteinase-12 (MMP-12).

In glioblastoma cells, CD133 expression has been confirmed to be significantly upregulated under hypoxic conditions. Furthermore, SCs marker expression in CD133^Neg^ non-CSCs changes under hypoxia, and turned cells into CD133^Pos^ CSCs, revealing that CSCs can be induced throughout dedifferentiation under hypoxic conditions [26]. These data shed light on new insights into tumor development, recurrence, and chemoradiotherapy resistance. Functional miRNAs can also alter CD133 expression in GSCs by regulating specific signaling pathways [27]. For instance, miR-181a inhibited the levels of CD133 and subsequently suppressed GSCs formation and glioblastoma tumorigenesis via the Notch-2 pathway [28]. Similarly, Chen et al. showed that miR-107 restrained CD133 expression by targeting the Notch-2 receptor and matrix metalloproteinase-12 (MMP-12) [29]. Another tumor suppressor miRNA in GSCs is miR-203. A study by Chen et al. [30] reported that miR-203 directly downregulated phospholipase D2 (PLD2). As a result, a reduction in self-renewal and proliferation of GSCs was observed. On the other hand, Deng et al. [31] observed that miR-203 expression was notably reduced in CD133^Pos^ GSCs derived from human glioblastoma biopsies. The expression of CD133 was significantly lowered in the miR-203-transfected CD133^Pos^ GSCs, and as such, the capacity for self-renewal was significantly reduced, possibly by a PLD2 reduction. CD133 also appears to be a target of miR-200b. miR-200b inhibited CD133^Pos^ GSCs’ stemness properties and division by targeting the PI3K/Akt pathway [32]. In harmony, other reports revealed that overexpression of miR-200b enabled the inhibition of stemness, proliferation, invasion, and migration of GSCs by targeting CD133 [33,34]. Conversely, miR-154 increased CD133 expression by inhibiting phosphoribosyl pyrophosphate synthetase 1 (PRPS1) in CD133^Pos^ GSCs [35], whilst miR-9 can activate the SHH signaling pathway by inhibiting protein patched homolog 1 (PTCH1), which enhanced the expression of CD133. Ultimately, this caused resistance to chemotherapy drugs like TMZ through the upregulation of multiple drug resistance 1 (MDR1) [36] (Figure 2).

Even though CD133 is known to be one of many markers that have been strongly linked to GSCs, controversy remains on the suitability of CD133 as a GSCs marker since CD133 is differentially glycosylated, resulting in mutable epitope masking [37]. Indeed, Barrantes-Freer et al. [38] revealed that CD133 expressed in GSCs is weakly immunoreactive for AC133. Moreover, the levels of CD133 on the surface of GSCs wiggle during the cell cycle, suggesting that CD133 is a marker of particular stages of division rather than a constituent marker of GSCs. In the context of conflicting reports and regarding the correlation between CD133 expression levels and the clinical pathological features and outcomes, Dahlrot et al. [39] concluded that overall survival and WHO grade did not correlate with CD133 expression status in gliomas. On the other hand, Han et al. [18] disclosed that patients with CD133^Pos^ had poorer progression-free survival (PFS) than patients with negative expression. They clarified the value of CD133 as a substantial clinical indicator for glioma patients with higher grade and worse prognosis. Therefore, the understanding of CD133 has opened a new avenue for therapeutic approaches in high-grade gliomas. 

### 2.2. CD44 

Another putative GSCs marker is CD44, which is a large cell adhesion molecule that acts as a receptor for hyaluronic acid (HA), a major element of the extracellular matrix [37,40]. There are multiple isoforms of CD44 that are produced by intermittent connections of a minimum of 10 exons and encoding fractions of the extracellular domain [41]. The brain is comparatively abundant in hyaluronic acid, suggesting that CD44 may be an important mediator of glioma cells’ migration inside the brain [42]. Among the various types of gliomas, CD44 is expressed with the highest expression of 55.55% in GBM [41]. 

A study scrutinizing the CD44 distribution in glioblastoma cells by Lim et al. [40] showed that CD44 cleavage and secretion take place at a massive rate on the leading edge of the tumor. Interestingly, standard CD44 (sCD44) was discovered in the opposite hemisphere of the brain, indicating that sCD44 is capable of propagating or circulating in the brain. sCD44 was shown to activate abnormal Tau pathology features, which indicates that sCD44 plays a pathological role between glioblastoma and neurodegeneration. 

The distribution of CD44 in glioblastoma cells were observed by Nishikawa et al. [43], who showed that elevated levels of CD44 are present within the invaded location at the tumor margin. Importantly, they discovered that GSCs with greater expression of CD44 in the tumor margin in comparison with the center correlates with highly invasive feature, shorter survival, and faster tumor progression. On the other hand, the migration and invasion of GSCs were considerably reduced by knockdown of the CD44 gene. This clearly indicates that CD44 has a basic role in tumor invasion and migration, thus supporting several reports that described CD44 as a marker of GSCs [41,44]. In contrast, one study found no significant correlation between CD44 expression and GSCs features. Wang et al. [45] showed that CD44 reduction under CD44 knockdown or HA-supplemented conditions augmented GSCs features, resulting in stimulation of GSCs markers’ expression, including Nestin, CD133, and OCT-4, as well as GSCs’ typical characteristics of sphere formation capability and long-term proliferation. This may suggest that CD44 is not an appropriate marker of GSCs. Instead, the main roles of CD44 may be proliferation, invasion, and migration.

It is noteworthy to mention that CD44 has shown a significant correlation with CD133 in GBM. It has been manifested that the proneural (PN) subtype of GSCs, but not mesenchymal (MES) GSCs preponderantly expresses CD133 while the MES subtype expresses the associated enrichment of CD44 [46,47]. These findings were confirmed by Brown et al. [48], who found a remarkable overlap of the CD133 marker with the PN subtype and conversely CD44 with the MES subtype. More recently, other reports have also clarified the relationship between these two markers and cell properties in GSCs. Cells expressing CD133 were found to be more proliferative, whereas cells expressing CD44 were more invasive [37,41]. Additionally, CD133 module signature (CD133-M) patients were observed to receive greater benefits from radiation treatment, whereas CD44 module signature (CD44-M) patients received higher benefits from chemotherapy with TMZ. This is probably because tumors with high expression of CD44 are more invasive, making them harder to be targeted by radiation therapy. In contrary, CD133 is involved in DNA replication and the cell cycle, hence making the CD133-M patient more vulnerable to drugs that target proliferative cells [48]. 

Hypoxia, as previously mentioned, has been shown to increase the expression of CD133 and promote GSCs’ proliferation. Brown et al. [37] explored the influence of hypoxia and TMZ on the CD44–CD133 equilibrium in GBM. Therein, they found that hypoxia induced a CD44^Pos^ to CD133^Pos^ shift, whereas TMZ caused the opposite shift. The ability of CD133^Pos^ cells to change their phenotype may be due to their active proliferation phenotype. Johansson et al. [44] investigated the involvement of CD44 in regulating hypoxic and pseudo-hypoxic signaling in GSCs. They found that the intracellular domain of CD44 (CD44ICD) is liberated in hypoxia, and subsequently bonded to the hypoxia inducible factor (HIF)-2α (but not HIF-1α). This improves HIF target gene activation, which is necessary for hypoxia-induced stemness in gliomas. Inhibiting CD44 cleavage at hypoxia caused a reduced HIF-2α level, but not HIF-1a. These data open the outlook of HIF-2α targeting via CD44. 

Another significant engagement of CD44 in enhancing tumor activities and progression is through the HA-mediated CD44 pathway by triggering intracellular miRNA and Rho GTPase signaling. Particularly miR-10b, which was overexpressed in malignant gliomas, together with overexpression of RhoC and uPAR contributed to invasion and migration [49]. Furthermore, HA/CD44 coordination regulates miR-21 production, which causes downregulation in the tumor suppressor protein of programmed cell death protein 4 (PDCD4). As a result, the expression of survival proteins (e.g., survivin, c-IAP1/2, and XIAP), stimulation of anti-apoptosis, and chemoresistance are enhanced in tumor cells [50]. Recent evidence revealed that overexpression of miR-373 in glioblastoma cells did not affect the cell growth but suppressed migration and invasion by inhibiting the expression of CD44 and TGFBR2, suggesting that CD44 can be directly targeted by miR-373 [51,52].

### 2.3. CD15

CD15 is a trisaccharide 3-fucosyl-N-acetyllactosamine, and is widely known as stage-specific embryonic antigen 1 (SSEA1). It has been confirmed to be prominently upregulated in various types of neutrophils and macrophages, and in several cancers [53,54,55]. In the adult brain, CD15 was shown to be remarkably expressed on pluripotent SCs and neural stem cells (NSCs), where it is believed to play a role in cell–cell interaction throughout neuronal growth [53,56]. PN subtype GSCs significantly express CD15 at the cell surface along with CD133 [47]. However, co-expression analysis revealed that CD15 is an MES marker, enriched in the MES subtype in an overlap with CD44, and therefore the usefulness of CD15 as a PN or MES marker remains open for further fulfillment [48].

The expression proportion of CD15 was found to be upregulated in non-CSCs under hypoxic conditions. Hypoxia actually induced sphere formation in glioblastoma-sorted non-CSCs and those newly formed spheres are highly expressed in SC markers, including CD15. This indicates that CD15^Pos^ GSCs can be stimulated throughout dedifferentiation under a hypoxic environment and this interchange between non-GSCs and GSCs perhaps promotes the cancer to become more malignant [26]. In the same manner of interconverting between non-GSCs and GSCs, Auffinger et al. [57] found that therapeutic doses of TMZ notably increased the expression of various glioma stem cell markers, such as CD15 and CD133, in vitro and in vivo. The finding was supported by William et al. [58], who then showed that glioblastoma cells, following constant TMZ exposure, can resume GSC properties, including an increase in the expression of CD133 and CD15. Similarly, the treatment with *N*-(p-coumaroyl) serotonin resulted in a noteworthy dose-dependent rise in the number of CD15 markers in GBM [59]. 

Although it was earlier believed to be a CSC marker in GBM, Kenney-Herbert et al. [56] showed that they could not isolate a genetically or phenotypically distinctive population for CD15. Furthermore, both CD15^Pos^ and CD15^Neg^ cells could generate mixed populations of glioblastoma cells and both were equally tumorigenic, with no survival advantage or early proliferative behavior for CD15^Pos^ cells. More recently, CD15^Pos^ cells were identified in both low- and high-grade gliomas. The expressions, however, were distinguishable in both grades, suggesting that CD15 might be useful to indicate tumor grades and survival rate [60]. Notwithstanding the controversy with respect to CD15’s precise role, little is known about its exact function in glioma.

### 2.4. CD70 (CD27L) 

CD70 is a type II transmembrane protein that belongs to the tumor necrosis factor (TNF) receptor family [61]. It is known as CD27 ligand (CD27L), another glycosylated transmembrane protein of TNF. So far, CD27 is the only ligand found that binds CD70 [62,63]. CD70, the receptor, is expressed on lymphomas and some other solid tumors, where it has a significant association with poor prognosis [64]. Its interaction with CD27 regulates cytotoxic T cells’ activity, which leads to prolonged survival in renal cell cancer mice [65]. Moreover, the interaction of T and B cells has been demonstrated to have immunosuppressive roles via T-cell apoptosis on the tumor microenvironment [66]. Likewise, but via M2 macrophages, the co-expression of CD70 and CD163 was found to be involved in poorer survival of GBM patients. These data imply that CD70 promotes tumor immunosuppression and aggressiveness through tumor-associated macrophage activation and recruitment [63]. 

Recent reports provided proof that CD70 expression was undetected in normal tissues from diverse organs; however, it was highly expressed in glioma tissues, indicating that CD70 expression is typically confined to tumors [61,62]. High-grade gliomas present robust expression of CD70 mainly in the GBM with the *IDH*-wild type variants, including epithelioid glioblastoma and gliosarcoma [61]. More specifically, CD70 is confirmed to be overexpressed in recurrent tumors and tumors with MES gene signatures of GBM and accordingly plays a role in the promotion of tumor migration [62]. Ablation of CD70 in glioblastoma cells reduced genes correlated with tumor epithelial mesenchymal transition (EMT), such as SOX-2 and CD44, and inhibited the migration and growth of the tumor [63]. 

Kitajima et al. [67] reported that in glioblastoma cells, HIF-2α upregulates and also causes the emergence of CD70 in CD70^Neg^ cells, while silencing HIF-2α resulted in a reduction of CD70 expression. Interestingly, knocking down HIF-1α did not considerably change CD70 expression in CD70^Pos^ cells. The loss of either CD70 or HIF-2α considerably weakened tumor growth, suggesting that both markers could be potential candidates for GBM therapy.

Jin et al. [62] demonstrated that targeting CD70^Pos^ glioblastoma cells with chimeric antigen receptor T cells (CAR T-cells) triggered a strong antitumor response, suggesting that CD70 is likely an optimal tumor immunotherapeutic target in GBM. This also indicates that CD70 could be used to increase positive outcomes, not only in primary glioblastoma patients but also in recurrent GBM that were found to be very highly expressing CD70. In addition, radiation enhanced CD70 expression on glioblastoma cells, offering good prospects to improve the antitumor efficiency to integrate standard care with CD70 CAR T-cell therapy. Due to the presence of the blood brain barrier in gliomas, CAR T-cell therapy could be a better approach as the activated T cells are not only capable of passing through the barriers but are furthermore able to induce a potential antitumor response [68]. However, one main drawback with T cells targeting overexpressing tumor receptors is the “on-target/off-tumor” toxicity against normal tissues. For instance, the engineered anti-CD19 CAR T cells have been used to successfully treat late-stage tumor patients with CD19^Pos^ B cell malignancies. However, it caused acute adverse effects, which were associated with increased cytokine release syndrome and long-term destruction of normal CD19^Pos^ B cells in some patients [69]. Therefore, further studies must be conducted to investigate whether these acute side effects can possibly be managed medically. 

### 2.5. S100A4 

S100A4, also known as FSP1/mts-1/metastasin/pEL98 [70], is a calcium-binding protein and EMT mediator [71,72]. This protein is an extensive spectrum trophic factor within the central nervous system, and it belongs to the S100 protein family [73,74]. It has been demonstrated that S100A4 is markedly overexpressed in the damaged human or rodent brain [75]. Pankratova et al. [73] showed that S100A4 binds to ErbB4 ligand and neuregulin (NRG), and that S100A4/ ErbB4/NRG signaling is crucial for neuroprotection in a damaged or injured brain. S100A4 was recognized as a prognostic marker, metastasis promoter, and regulator in several cancers, including glioblastoma [70], head and neck cancers [71], and colorectal cancer [76]. 

Liang et al. [77] confirmed that glioma progress with MES features was partially mediated by S100A4. A recent report provided more robust clarification and definitive proof for the roles of S100A4 as a critical regulator and a novel marker of GSCs. It was shown that S100A4^Pos^ cells were capable of initiating a tumor and forming spheres, and that S100A4 is essential to maintain self-renewal for GSCs. Whilst the molecular mechanism that supports the GSCs in self-renewal and maintenance still needs to be clarified, it seems that S100A4 is involved in the upstream processes of EMT and MES transition [70]. S100A4 depletion lengthened the anti-VEGF treatment profile and hence reduced glioblastoma resistance to antiangiogenic therapy. Therefore, the targeting of S100A4 may be a promising approach to constrain glioma malignancy [77,78]. In addition, Aguilar-Morante et al. [79] showed that CCAAT/enhancer-binding protein β (C/EBPβ) suppression substantially decreased the levels of S100A4 in glioblastoma cells, resulting in inhibitions to growth, transformation capacity, and migration. Thus, targeting C/EBPβ in glioblastoma cells is therapeutically strategic to block the S100A4 gene. 

### 2.6. ALDH1A3 

Aldehyde dehydrogenases (ALDHs) are a group of enzymes of one class that metabolize endogenic and exogenic aldehydes to their respective conforming carboxylic acids [80]. High ALDH activity, predominantly the members of ALDH1, has been identified in CSCs of different tumors, including breast, lung, sarcoma, and gastric cancer [81]. ALDH1A3 in particular has been indicated as a marker that promotes GSCs and correlates with the MES phenotype and invasion in human glioblastoma [82,83,84]. However, gliomas with low expression of ALDH1A3 were more likely to be the PN subtype [84], and suppression of ALDH1A3 inhibited PN GSCs’ proliferation [85]. Li et al. [86] furthermore confirmed that ALDH1A3 is not only associated with the MES lineage of GBM but is also the key driver of enhancing MES subtype differentiation.

Recent work by Cheng et al. [83] showed that the transcription factor FOXD1 directly controls the transcriptional function of ALDH1A3. They defined FOXD1-ALDH1A3 signaling as a vital pathway in the self-renewal and tumorigenicity of MES GSCs. Hence, it provides a potential new molecular target for treating GBM. Another ALDH1A3-associated signaling pathway was reported by Sullivan et al. [87], where it promotes stem cell-like properties by inducing the expression of tissue transglutaminase (tTG), an enzyme previously linked to the initiation and progression of aggressive tumors. In glioblastoma cells, tTG contributes to the aggressiveness of MES GSCs by stimulating proliferation, self-renewal, and survival. The inhibition of this enzyme with TMZ or radiotherapy impaired proliferation and enhanced cell death. These results ascribe a novel function for ALDH1A3 in an aggressive MES GSCs phenotype via the upregulation of tTG. 

Zhang et al. [84] evaluated the relationship between ALDH1A3 expression and clinical outcome and found that ALDH1A3 was significantly overexpressed in high-grade gliomas in comparison with low-grade gliomas. They also found a higher mortality and a worst overall survival associated with overexpression of ALDH1A3. Besides, cell invasiveness was reduced when ALDH1A3 was knocked down. Wu et al. [81] confirmed that ALDH1A3 knockdown in glioblastoma cells resulted in more sensitivity towards TMZ. In wild-type cells, the expression of ALDH1A3 was diminished with rising concentrations of TMZ up to ≥300 µM. This inhibition was found to be due to a direct interaction with p62, an autophagy adaptor protein, resulting in the downregulation of ALDH1A3 by autophagy, whilst the inhibition of autophagy led to ALDH1A3 accumulation. Similarly, ALDH1A3 has been reported to be downregulated in response to the combination of focal adhesion kinase (FAK) autophosphorylation inhibitor, Y15, and TMZ, indicating a synergistic contribution upon treating drug resistance [88]. Collectively, these data suggest that ALDH1A3 can serve as a prognostic biomarker of gliomas, and as one of the leading targets for more effective therapies for MES-subtype gliomas.

### 2.7. NANOG

Nanog is a homeodomain transcription factor that controls the expression of a horde of downstream genes and is widely known to be involved in the regulation and maintenance of embryonic stem cells’ (ESCs) pluripotency [89]. The Nanog gene is positioned on chromosome 12 and is named after the fountain of youth in celtic mythology, Tir Na Nog [90]. Numerous studies have long-established that Nanog is a CSC surface marker in tumors [91], including glioma [92], and its expression has been found to be correlated with gender, differentiation, depth of infiltration, and the TNM classification of malignant tumors (TNM stage) [91]. 

Nanog has been established as a novel hedgehog (HH) and glioma-associated oncogenes (GLI) signaling pathway mediator essential for glioblastomas. Depending on the HH signaling pathway, GLI directly bind to the Nanog promoter and the GLI-Nanog axis mediates stemness and GSCs progression [93]. More recently, Kakiuchi et al. [94] observed the same trends in acute myeloid leukemia (AML) SCs that overexpress Nanog. They also reported that Nanog could induce quiescent SCs towards cell cycling in leukemia and other cancers that express high levels of Nanog.

High expression levels of Nanog were associated with shorter survival in low- and high-grade gliomas patients, indicating that it is significantly attributed to the clinical outcome of gliomas [95]. Niu et al. [96] reported a positive pathological-grade correlation with Nanog and CD133 co-expression. They demonstrated that Nanog overexpression and its close relationship with the undifferentiated state of glioblastoma contributed to tumorigenesis by the maintenance of its undifferentiated state. On the basis of their findings, they assumed that Nanog inhibition may block the tumorigenesis of glioblastoma, and targeting Nanog may be an effective approach to enhance the therapeutic intervention for poorly differentiated glioblastoma. Likewise, Nanog and CD133 were shown to have a positive relationship under hypoxic conditions. In addition to CD133, Nanog was consistently upregulated by hypoxia in cell populations obtained from glioblastoma patients [97].

To clarify whether Nanog is critical for GBM prognosis, Soni et al. [98] showed that Nanog is augmented by CD24 expressing the SCs gene that correlated with the increase of lymph nodes and distant metastasis. Cases with higher expressions of CD24 and Nanog had significantly poorer survival. However, Bien-Möller et al. [99] reported that Nanog was markedly upregulated in stem-like neurospheres in glioblastoma, but its expression was not correlated with the patient survival period. In a recent study, Nanog mRNA expression levels were significantly downregulated compared to non-cancerous tissues in a GBM patient [100]. These findings may appear to dispute the stemness role of this pluripotent transcription factor that comes into play. In contrast, Zbinden et al. [101] found that Nanog was in fact essential for glioblastoma tumorigenicity. 

By mediation of miR-137, downregulation of Nanog together with OCT-4 and SOX-2 expressions increased differentiation and decreased proliferation in glioblastoma stem cells. These findings confirm the important role of Nanog in the stemness of GSCs [102]. In addition, miR-134 expression is a common event in gliomas [103]. Niu et al. [104] reported that miR-134 overexpression in glioblastoma inhibited proliferation and invasion by inhibiting Nanog expression, confirming that Nanog is also significantly correlated with glioblastoma invasiveness and migration. 

### 2.8. OCT-4

Octamer-binding protein transcription factor 4 (OCT-4), a part of the Pit-Oct-Unc (POU) family, is another pluripotency factor that has been proposed to be a tumor-initiating stem cell (TISC)-related marker. Expression of OCT-4 was found in ESCs and germ cells, responsible for the pluripotency and self-renewal of SCs and involved in differentiation regulation [105]. In 1991, OCT-4 mRNA and protein were for the first time discovered in oocytes before and after fertilization. The OCT-4 gene is localized on chromosome 6P21.3 and OCT-4 protein is encoded by POU5F1 [106]. 

All astrocytic brain tumors showed some level of OCT-4 expression. Together with GLI1, OCT-4 has a transcriptional regulation mechanism of secreted phosphoprotein 1 (SPP1). In glioma-initiating cells and glioblastoma, both OCT-4 and GLI1 are overexerted and this axis maintains the stemness phenotype by binding to the SPP1 gene [107]. GLI1 belongs to the SHH pathway and is overexpressed in glioblastoma tumors. It exhibits overexpression not only for OCT-4 but also for Nanog and SOX-2, whilst its inhibition downregulated OCT-4 and Nanog. GLI1 activation was shown to be mediated by the Akt pathway when Ranjan et al. [108] confirmed glioblastoma growth suppression through GLI1 inhibition. Furthermore, Li et al. [109] confirmed the crosstalk between Akt and OCT-4. They demonstrated that Akt could obliquely regulate the mRNA levels, transcriptional activities, and protein stability of OCT-4 in ESCs. Other studies have shown that OCT-4 knockdown in embryonic carcinoma cells augmented Akt expression levels, whereas blocking of the Akt pathway enhanced the expression of OCT-4 in GSCs [110,111]. These results indicate a negative regulation relationship between Akt and OCT-4. 

The hallmark of glioblastoma is known to be the existence of hypoxia with an abnormal vascular supply. At severe hypoxia, EMT and stemness markers, including OCT-4, are upregulated by the FAT1 gene, an orthologous of the *Drosophila* tumor suppressor gene *fat* GBM tumors. FAT1 knockdown in glioblastoma inhibited all EMT and stemness markers, including OCT-4 [112]. Similarly, Bhagat et al. [113] reported that the expression of all invasive factors and stemness factors, including OCT-4 and SOX-2, is regulated under hypoxia. They showed that a HIF-2α-SOX-2/OCT-4-Mena axis is intensely activated in hypoxia and significantly increased the migratory potential of the glioblastoma cells. OCT-4^Pos^ GSCs also showed a significant positive correlation with nucleolin, which was found to be involved in promoting tumor growth in GSCs. Nucleolin was suggested as a potential therapeutic marker in OCT-4^Pos^ GSCs, and therefore targeting this protein can perhaps diminish stemness and cell aggressiveness [114]. Additionally, OCT-4^Pos^ cells have been shown to be positively correlated with tumor grade and malignancy in GBM; however, no association between prognostic influence and OCT-4^Pos^ cells was identified [105]. 

A group of miRNAs, including miR-20a, miR-20b, miR-106a, miR-106b miR-145, and miR-335, was accomplished by regulating OCT-4 [115]. For instance, miR-145, a tumor suppressor and a repressor of pluripotency in ESCs, was found to be downregulated in glioblastoma and GSCs [116]. Indeed, Yang et al. [117] showed that miR-145 expression is inversely correlated with OCT-4 and SOX-2 levels in CD133^Pos^ GSCs. This indicates that this miRNA has an important role in suppressing tumorigenic, self-renewal, and chemo/radioresistance in GSCs by targeting the downstream of the stemness genes OCT-4 and SOX-2. Similarly, Gao et al. [118] showed that the overexpression of miR-141 exhibited downregulation of both of the co-upregulated genes, EMT, and stemness genes, including OCT-4. Therefore, miR-141 might serve as an effective antioncomiR targeting in OCT-4^Pos^ GSCs. 

### 2.9. SOX-2

SOX-2, sex-determining region Y (SRY)-box 2, belongs to the sry-related high-mobility group (HMG) box (SOX) family of transcription factors [119]. SOX-2 was discovered in 1994 and is situated on chromosome 3q26.3-q27 and encrypts a protein involving 317 amino acids [120]. SOX2, alongside other components of its network (OCT-4 and Nanog), promotes SCs’ pluripotency [121]. SOX-2 has been evidenced to be abnormally expressed in a range of solid tumors, such as prostate cancer, lung cancer, breast cancer, glioblastomas, and melanomas [122]. In addition, protein SOX-2 has been shown to play a role in metastasis, proliferation, apoptosis, tumorigenesis, and invasion of various cancer cells [123].

In glioma, SOX-2 expression is frequently high and has been found to be critical for growth and survival and is closely related to the relapse after chemotherapy or radiotherapy [124]. Garros-Regulez et al. [125] reported that SOX-2 inhibition prompts cellular senescence in differentiated glioblastoma cells. Moreover, they showed that overexpression of SOX-2, in addition to promoting invasiveness and migration, is essential for GSC maintenance. They showed that cells with high expression of SOX-2 are more resistant to TMZ, assuming SOX-2 as one of the key proteins responsible for resistance to chemotherapy in GBM. Another report confirmed that SOX-2 correlated significantly with treatment resistance. In CD133^Pos^ GSCs, SOX-2 protein has been recognized as one of the CD133 downstream targets. The alliance of CD133^Pos^ and SOX-2 is suitable for therapeutics targeting glioblastoma because of the critical role it plays in GSCs maintenance, causing resistance to chemotherapy and radiotherapy [126]. Additionally, SOX-2 has shown distinct roles for self-renewal in GSCs by its interaction with FOXG1, a member of the fork head box family of transcription factors and one of the most overexpressed genes in glioblastoma [127]. 

Dong et al. [120] carried out an experiment to confirm whether SOX-2 was a direct target of miR-429, a member of the miR-200 family that has been found to act as either oncogenes or tumor suppressors in glioblastoma. They showed that miR-429 applies a preventive influence on the propagation and invasion of glioblastoma cells by directly targeting SOX-2. Luo et al. [128] discovered that miR-126-3p sensitized glioblastoma cells to TMZ by targeting SOX-2-Wnt/β-catenin. Their findings showed that miR-126-3p downregulates SOX-2 expression and thus blocks the Wnt/β-catenin pathway. Likewise, another study demonstrated that miR-145 enhanced the chemosensitivity of GSCs upon desmethoxycurcumin (DMC) also by targeting SOX-2-Wnt/β-catenin [129]. Elsewhere, GSCs’ state features and maintenance were shown to be inhibited due to the repression of SOX-2 and Nanog by miR-34a. Furthermore, SOX-2 was found to be a direct target of miR-124, causing slower migration and self-renewal in GSCs [130]. 

### 2.10. NESTIN

Nestin is an intermediate filament (IF) protein (type VI) consisting of 1621 amino acids to produce a molecular weight of 177.4 kDa protein [131]. Following the identification of this protein in 1985, many studies have been conducted on the biological role of Nestin. It was firstly defined as a neuronal stem/progenitor cell marker and identified to be participating in cytoskeletal organization [132]. According to Park and his colleagues, the presence of Nestin is essential for the self-renewal of NSCs [133]. Recently, Nestin has been identified as endothelial cell enriched through all adult vascular beds. Thus, challenging the doctrine that Nestin is confined to locations of tissue regeneration and showing that it is an essential body-wide protein [134]. In tumors, Nestin expression is not limited to cancer cells but also occurs in newly forming tumor vessels [135], and is a valuable marker of ongoing angiogenesis and CSCs [136]. In some tumors, high expression of Nestin has is correlated with metastasis and aggressive growth. This was observed in several cancers, including GBM [137]. 

In glioblastoma cells, Nestin regulates growth, stemness, and invasion through the alteration of HSC71 (gene HSPA8). As such, inhibition of Nestin and/or HSC71 may be a beneficial molecular target therapy for glioblastoma [138]. Many reports have showed that Nestin knockdown suppressed the proliferation, invasion, and migration of glioblastoma cells [139,140]. Some studies suggested that Nestin expression is linked to a higher grade and worsens prognosis in gliomas. For example, Strojnik et al. [141] demonstrated that Nestin expression was a very strong prognostic marker for high-grade gliomas with poor prognostic outcome. Similarly, Arai et al. [142] confirmed that Nestin is a beneficial marker for the diagnosis of high-grade gliomas due to the significant positive relationship between its expression and poor prognosis. Moreover, Lv et al. [143] concluded that higher Nestin expression is associated with higher grade gliomas and that Nestin can be used as an overall survival (OS) and PFS prognostic indicator associated with poor clinical pathological features. 

Nestin^Pos^ GSCs proliferation is promoted by the activation of the KRAS/Notch pathway, whereas resistance to radiotherapy has been exhibited by the activation of the Akt/PI3K and p53 pathways [144]. miR-381 suppression has been sensitized by glioblastoma cells to TMZ by preventing stemness factors, including Nestin [145]. Additionally, miR-423-5p through suppression of its target gene (ING-4) increased Nestin expression in GSCs and accordingly induced glioblastoma cells to exhibit greater resistance to TMZ [146]. Therefore, the prevention of miR-423-5p or miR-381 together with TMZ intervention may be a beneficial therapeutic approach for suppressing GSCs growth. 

## 3. Conclusions

**Figure 3:**
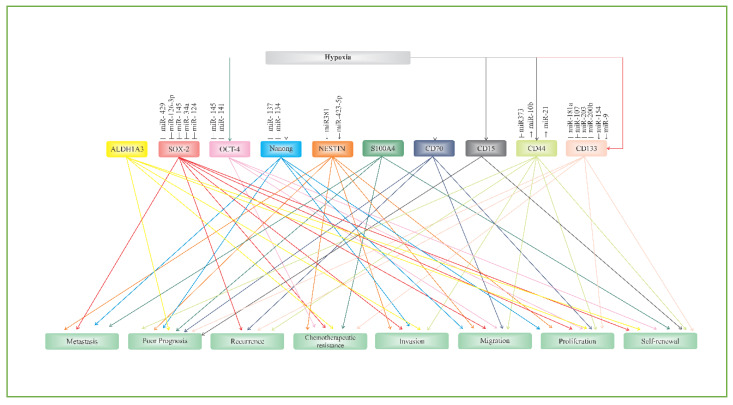
Biomarker/miRNAs network axes in glioblastoma signaling pathways.

In neurooncology, glioblastoma, otherwise known as GBM, remains a conundrum. Intensive multimodal treatments, such as surgery, radiotherapy, and chemotherapy, have failed to significantly improve the median survival of patients. One of the reasons that may have contributed to this failure is the complex communication network of putative glioblastoma stem cell (GSC) biomarkers. Tremendous efforts to develop a novel treatment that is specific for GSCs are ongoing. Some researchers are focusing on developing a new approach that can efficiently kill GSCs, which are responsible for poor prognosis, proliferation, migration, invasion, and chemotherapeutic resistance of GBM, while some groups are using modern molecular biology tools, such as miRNAs, to knockdown tumor growth and survival. Elucidating the biological nature of GSCs may offer a new strategy for targeted GBM therapy. Therefore, understanding the communication network axis of biomarker–biomarker, biomarker–signaling molecules, and biomarker–miRNAs plays a pivotal role in constructing future GBM treatments that can move the needle (Figure 3).
